# Repurposing antihypertensive drugs for pain disorders: a drug-target mendelian randomization study

**DOI:** 10.3389/fphar.2024.1448319

**Published:** 2024-08-29

**Authors:** Kai Du, Ao Li, Chen-Yu Zhang, Shu-Ming Li, Ping Chen

**Affiliations:** ^1^ Graduate School, Beijing University of Chinese Medicine, Beijing, China; ^2^ Department of Pain Medicine, Beijing Hospital of Traditional Chinese Medicine, Capital Medical University, Beijing, China

**Keywords:** antihypertensive drug, hypertension, pain management, drug target mendelian randomization, drug repurposing

## Abstract

**Objective:**

Addressing the rising prevalence of pain disorders and limitations of current analgesics, our study explores repurposing antihypertensive drugs for pain management, inspired by the link between hypertension and pain. We leverage a drug-target Mendelian Randomization (MR) approach to explore their dual benefits and establish causal connections.

**Methods:**

A comprehensive compilation of antihypertensive drug classes was undertaken through British National Formulary, with their target genes identified using the DrugBank database. Relevant single nucleotide polymorphisms (SNPs) associated with these targets were selected from published genomic studies on systolic blood pressure (SBP) as genetic instruments. These SNPs were validated through MR against acute coronary artery disease (CAD) to ensure genes not linked to CAD were excluded from acting as proxies for antihypertensive drugs. An MR analysis of 29 pain-related outcomes was conducted using the FinnGen R10 database employing the selected and validated genetic instruments. We utilized the Inverse Variance Weighted (IVW) method for primary analysis, applying Bonferroni correction to control type I error. IVW’s multiplicative random effects (MRE) addressed heterogeneity, and MR-PRESSO managed pleiotropy, ensuring accurate causal inference.

**Results:**

Our analysis differentiates strong and suggestive evidence in linking antihypertensive drugs to pain disorder risks. Strong evidence was found for adrenergic neuron blockers increasing migraine without aura risk, loop diuretics reducing panniculitis, and vasodilator antihypertensives lowering limb pain risk. Suggestive evidence suggests alpha-adrenoceptor blockers might increase migraine risk, while beta-adrenoceptor blockers could lower radiculopathy risk. Adrenergic neuron blockers also show a potential protective effect against coxarthrosis (hip osteoarthritis) and increased femgenpain risk (pain and other conditions related to female genital organs and menstrual cycle). Additionally, suggestive links were found between vasodilator antihypertensives and reduced radiculopathy risk, and both alpha-adrenoceptor blockers and renin inhibitors possibly decreasing dorsalgianas risk (unspecified dorsalgia). These findings highlight the intricate effects of antihypertensive drugs on pain disorders, underlining the need for further research.

**Conclusion:**

The findings indicate that antihypertensive medications may exert varied effects on pain management, suggesting a repurposing potential for treating specific pain disorders. The results advocate for further research to confirm these associations and to explore underlying mechanisms, to optimize pain management practices.

## 1 Introduction

The global prevalence of pain poses significant challenges to public health, impairing quality of life and imposing socioeconomic burdens (Zimmer et al., 2022). The International Association for the Study of Pain (IASP) defines pain as an unpleasant sensory and emotional experience linked to actual or potential tissue damage (Raja et al., 2020). Chronic pain, affecting 20%–30% of the global population, varies in prevalence across regions and populations, with acute pain responding directly to injury or illness and chronic pain persisting and often associated with conditions like arthritis, cancer, and neuropathy. Pain’s association with psychological issues such as depression and anxiety complicates its management (Cieza et al., 2020).

Current pain treatment methods, including pharmacological interventions, physical therapy, and psychological support, face limitations in long-term chronic pain management, highlighting the need for novel therapeutic strategies. Drug repurposing, the investigation of approved medications for new therapeutic indications, emerges as a promising approach, potentially offering safer, more effective treatment options for pain sufferers. Notably, the significant negative impact of hypertension on conditions such as back pain, spinal pain, neck pain, lumbar spine degeneration, osteoarthritis (OA), and fibromyalgia presents a unique opportunity for the application of antihypertensive drugs in pain management (Kauppila, 2009; Williams et al., 2018; Allen et al., 2022; Ahorukomeye et al., 2023; Suri et al., 2023). Antihypertensives, especially beta-adrenoceptor blockers (BBs), have demonstrated significant efficacy in reducing perioperative pain, minimizing long-term opioid use, and alleviating the risks associated with knee and hip OA (Härkänen et al., 2015; Nakafero et al., 2019; Nakafero et., 2021; Starr et al., 2019; Zhou et al., 2020; Li et al., 2021). Furthermore, BBs have shown potential in relieving injection site pain, chronic tension-type facial pain, fibromyalgia, and temporomandibular joint disorders, underscoring the potential role of antihypertensive drugs in pain management and providing a scientific basis for their clinical application (Yavascaoglu et al., 2007; Kruger and Light, 2010; Tchivileva et al., 2010; Agius et al., 2013; Akgün Salman et al., 2013; Ergil et al., 2015). BBs and calcium channel blockers (CCBs) have also shown potential therapeutic effects for migraine and cluster headaches (Tfelt-Hansen and Tfelt-Hansen, 2009; Jackson et al., 2015; 2019). While certain limitations exist, the use of antihypertensive medications may offer a safer, more effective treatment option for pain sufferers.

Preliminary studies hint at the potential of antihypertensive medications to manage pain disorders effectively, but various factors limit the reliability of such findings. Challenges in observational studies, including confounding variables and the inability to establish causality, along with variability in study design, dosage, and population characteristics, contribute to inconsistent results. The complexity of pain, influenced by genetic, environmental, and psychosocial factors, further complicates result interpretation. Additionally, discrepancies across studies raise questions about the generalizability and real-world applicability of these findings (Zhou et al., 2020; Li et al., 2021; Nakafero et al., 2021). These issues highlight the necessity for robust methodologies like Mendelian Randomization (MR), offering a path to more reliable evidence on how antihypertensive drugs might affect pain management.

MR offers a sophisticated approach to establish causality in epidemiological studies by using genetic variants, specifically single nucleotide polymorphisms (SNPs), identified through Genome-Wide Association Studies (GWAS) as instrumental variables (IVs). This method effectively simulates the randomization process found in controlled trials, providing a robust framework for overcoming the limitations often encountered in traditional observational and experimental research. By leveraging genetic predispositions that are randomly assigned at conception, MR controls for confounding variables and minimizes the risk of reverse causation, thereby offering more accurate estimates of the causal effects of antihypertensive drug use on pain reduction. Utilizing the power of MR, this study aims to clarify the causal relationship between antihypertensive drug use and pain conditions. The significance of this research lies in its potential to inform safer and more effective treatment strategies for individuals suffering from pain disorders, thereby improving patient outcomes and quality of life.

## 2 Materials and methods

### 2.1 Study design and assumptions

**FIGURE 1:**
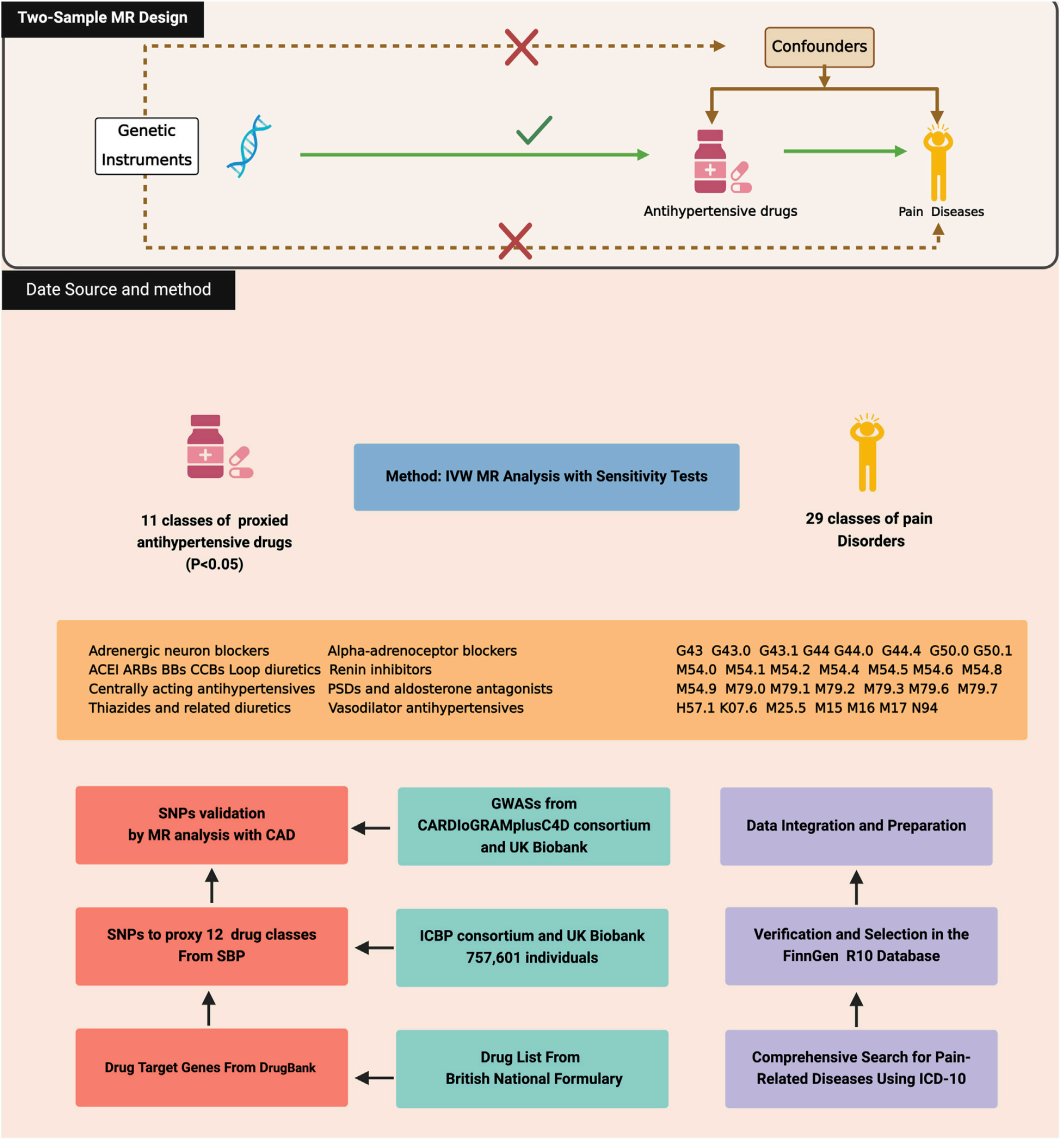
Three assumptions of Mendelian randomization and the study design of this study, created with BioRender.com. CAD, Coronary Artery Disease; ACEI, Angiotensin converting enzyme inhibitors; ARBs, Angiotensin-II receptor antagonists; BBs, Beta-adrenoceptor blockers; CCBs, Calcium channel blockers.

This study systematically explores the use of antihypertensive drugs for pain management through the MR framework, focusing on genetic variations associated with blood pressure regulation as a proxy for drug efficacy. We meticulously compiled a list of antihypertensive drug classes from the British National Formulary, mapping them to target genes through the DrugBank database. Relevant SNPs linked to systolic blood pressure (SBP), drawn from genomic studies, serve as genetic instruments for analyzing the effects of twelve primary antihypertensive classes. These SNPs were validated against acute coronary artery disease (CAD) to ensure specificity to antihypertensive effects. Utilizing the FinnGen R10 database, our analysis covers 29 pain-related outcomes, assessing the potential efficacy of antihypertensive medications across diverse pain conditions. This approach not only mitigates confounding factors but also provides a robust basis for evaluating the causal relationships between antihypertensive medication use and pain conditions, thereby contributing to the development of personalized medicine and targeted treatments for pain disorders. In adhering to the methodological rigor required for the MR framework, this study ensures that the three core assumptions underpinning MR are met. First, the genetic variations associated with blood pressure, which serve as proxies for the efficacy of antihypertensive drugs, are valid IVs, having a direct and strong association with the exposure of interest as evidenced by their linkage to SBP. Second, the selection of SNPs was conducted with an emphasis on independence from confounders, a process bolstered by their validation against CAD to ensure the observed associations with pain outcomes are not confounded by cardiovascular disease. Third, the exclusion restriction assumption is satisfied, as the analysis is designed to capture the effects of the SNPs on pain through their impact on drug response only, with sensitivity analyses employed to check for and address any potential pleiotropic effects. Figure 1 visually summarizes the study design and assumptions (Birney, 2022).

### 2.2 Data sources and populations

#### 2.2.1 Genetic associations with antihypertensive drugs

Building upon the design framework and Genetic instruments from prior research (Wang et al., 2023), this investigation meticulously cataloged a comprehensive spectrum of twelve antihypertensive drug classes as delineated by the British National Formulary, inclusive of adrenergic neuron blockers, alpha-adrenoceptor blockers, angiotensin-II receptor antagonists (ARB), angiotensin converting enzyme inhibitors (ACEI), BBs, CCBs, centrally acting antihypertensives, loop diuretics, PSDs and aldosterone antagonists, renin inhibitors, thiazides and related diuretics, and vasodilator antihypertensives. Using the DrugBank database, target genes for the active ingredients within these classes were pinpointed (Supplementary Table S1). After identifying target genes, SNPs associated with SBP were selected from GWAS meta-analysis by the International Consortium of Blood Pressure (ICBP) and the UK Biobank. This choice was informed by SBP’s relevance as a primary target for antihypertensive drugs, utilizing these SNPs as proxies to investigate their impact on pain disorders (Evangelou et al., 2018). This selection adhered to stringent criteria, including a genome-wide significance threshold (*p* < 5 × 10^−8^), a proximity requirement (±100 kb) to the drug target genes, and low linkage disequilibrium (LD) (r^2^ < 0.1) (Yarmolinsky et al., 2022). When primary target SNPs were unavailable, alternative SNPs exhibiting high linkage disequilibrium (r^2^ > 0.80), indicative of a strong genetic association, were identified using LDlink. This approach ensures the comprehensive inclusion of relevant genetic variants. To enhance the reliability of our genetic analysis, the study excluded SNPs with ambiguous or palindromic sequences and employed a harmonization process across all datasets. This critical step ensured that our effect estimates remained consistent and robust.

#### 2.2.2 Genetic associations with CAD and pain

CAD was utilized as a control outcome to validate the effectiveness of our selected SNPs, given the recognized critical role of antihypertensive medication in reducing CAD morbidity rates. The CAD data were derived from a GWAS conducted by the CARDIoGRAMplusC4D consortium and the UK Biobank, comprising 122,733 cases and 424,528 controls (van der Harst and Verweij, 2018). The study also analyzed 29 pain-related outcomes using the FinnGen R10 database, which includes data on 230,310 females and 181,871 males, covering over 21 million genetic variants across 2,408 conditions (Kurki et al., 2023). This analysis was guided by the International Classification of Diseases, Tenth Revision (ICD-10), ensuring a thorough examination of various pain conditions and their genetic associations. The conditions studied ranged from migraines and headache syndromes to trigeminal nerve and brachial plexus disorders, among others, with a detailed listing in Supplementary Table S2. Utilizing ICD-10 for disease categorization within the FinnGen R10 database enhanced the uniformity and comparability of data while integrating genetic information with comprehensive health records laid a solid foundation for our MR analysis. This methodology facilitated the exploration of genetic links to a wide spectrum of clinical outcomes, providing insights into the genetic basis of pain disorders and their potential treatment with antihypertensive medications.

### 2.3 Statistical analysis

Genetic proxies for antihypertensive drugs were carefully chosen based on their significant association with SBP and validated through their relevance to CAD risk reduction, employing the Inverse variance weighted (IVW) method to ensure these instruments’ applicability. Subsequently we took the IVW approach to test the association between valid genetically antihypertensive drugs and pain disorders (Bowden et al., 2015; Slob and Burgess, 2020). Confronted with the complexity of assessing numerous antihypertensive drug classes against a unified pain outcome, our study mandated a strict multiple-testing regimen to maintain statistical rigor. The application of the Bonferroni correction, setting a refined significance cut-off at *p* < 0.0042, was crucial to effectively distinguish between conclusive and suggestive evidence (*p* values from 0.0042 to <0.05) (Curtin and Schulz, 1998). This stringent approach was instrumental in facilitating the reliable estimation of the odds ratio (OR) for a 1 mmHg decline in SBP attributable to the use of antihypertensive drugs, enhancing the precision of our OR and confidence interval (CI) estimates. Robust IVs were identified through detailed calculations of PVE and F-statistics, ensuring the selection of genetic instruments with strong and reliable associations with antihypertensive drug efficacy (Burgess et al., 2011; Brion et al., 2013).

### 2.4 Sensitivity analysis

Integral to our methodological framework was a thorough sensitivity analysis. This critical component not only validated the consistency of our results but also reinforced the reliability of our causal conclusions. Given the challenge posed by heterogeneity—differences in causal estimates across IVs—our analysis incorporated multiplicative random effects (MRE) model within the IVW framework (Greco M et al., 2015). This approach was instrumental in bolstering the strength of our findings by accommodating variance among different IVs, thereby enhancing the reliability of our causal deductions. Additionally, horizontal pleiotropy, where IVs might affect the outcome through pathways not directly related to the exposure, posed a significant threat to the integrity of our causal deductions. To counteract this, we employed MR Egger regression, allowing for a quantitative adjustment for pleiotropic effects, alongside the MR Pleiotropy RESidual Sum and Outlier (MR-PRESSO) test to identify and exclude pleiotropic outliers (Verbanck et al., 2018). The integrity of our causal conclusions was also scrutinized using the leave-one-out method, ensuring that no individual IV disproportionately influenced the overall results. Visual diagnostics, including scatter, funnel, and forest plots, were employed to demonstrate our comprehensive efforts in addressing pleiotropy and maintaining the rigor of our analysis. To ensure the specificity and relevance of our instrumental variables, we meticulously examined the associations between SNPs related to the efficacy of antihypertensive drugs and various potential confounders linked to pain disorders through the Phenoscanner platform. This crucial scrutiny, guided by a stringent linkage disequilibrium threshold and alignment with the Human Genome Reference Assembly build 37 (GRCh37/hg19), helped in filtering out SNPs associated with confounding factors such as insomnia, anxiety, obesity, alcohol intake, and smoking, thereby reducing the influence of horizontal pleiotropy.

Furthermore, the MR Steiger test validated the directionality of associations between SNPs and the antihypertensive drug targets, ensuring that the SNPs exerted a stronger influence on the exposure than on the outcome. This validation, supported by Steiger *P*-values below 0.05 and an evaluation of the test’s power, refined our selection of genetic instruments, enhancing the study’s precision in delineating causal relationships (Hemani et al., 2017).

The MR analysis conducted in this investigation made use of specific software packages within the R software environment (version 4.3), notably TwoSampleMR (version 0.5.7) for executing TSMR analyses, and MRPRESSO (version 1.0) for the detection and correction of pleiotropic outliers. Furthermore, the reporting of our MR methodology adheres to the STROBE-MR statement, thereby guaranteeing transparency and methodological rigor.

## 3 Results

### 3.1 Genetic instrument selection

**FIGURE 2:**
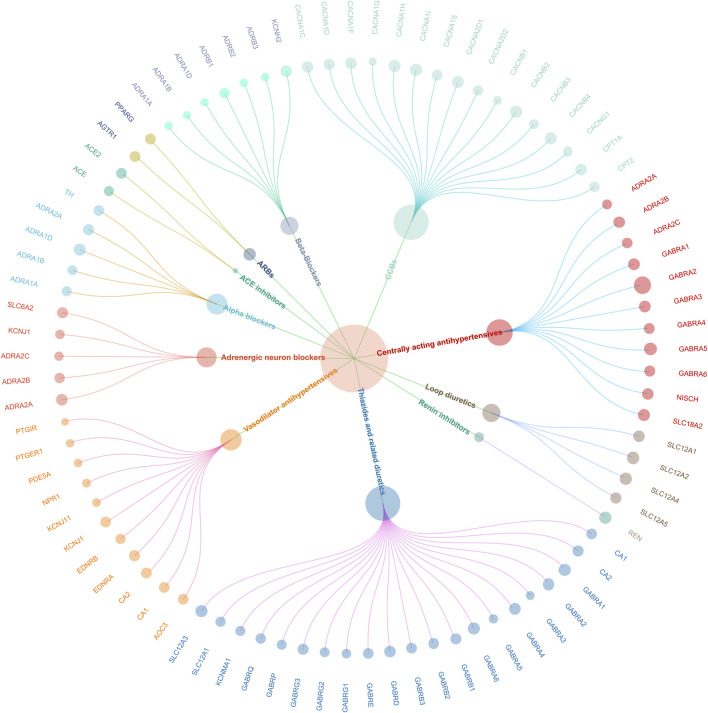
Antihypertensive drug classes and associated genes post-CAD validation.

In the study, 464 SNPs associated with SBP were utilized to create genetic proxies for evaluating the effects of 12 antihypertensive drug classes on cardiovascular health and pain disorders (Supplementary Table S3). Through MR analysis, significant associations were identified for 11 of these classes in relation to CAD, except for PSDs and aldosterone antagonists. This finding indicates a nuanced interaction between antihypertensive treatments and CAD risk, detailed in Supplementary Table S4. The validity of these genetic instruments was confirmed by F-statistics greater than 10, showcasing the MR methodology’s rigor. A genetic map illustrating the connections between these 11 drug classes and their genetic markers is presented in Figure 2, providing a visual representation of the study’s foundational genetic analysis.

### 3.2 Strong evidence for the association between antihypertensive drugs and pain disorders

**FIGURE 3:**
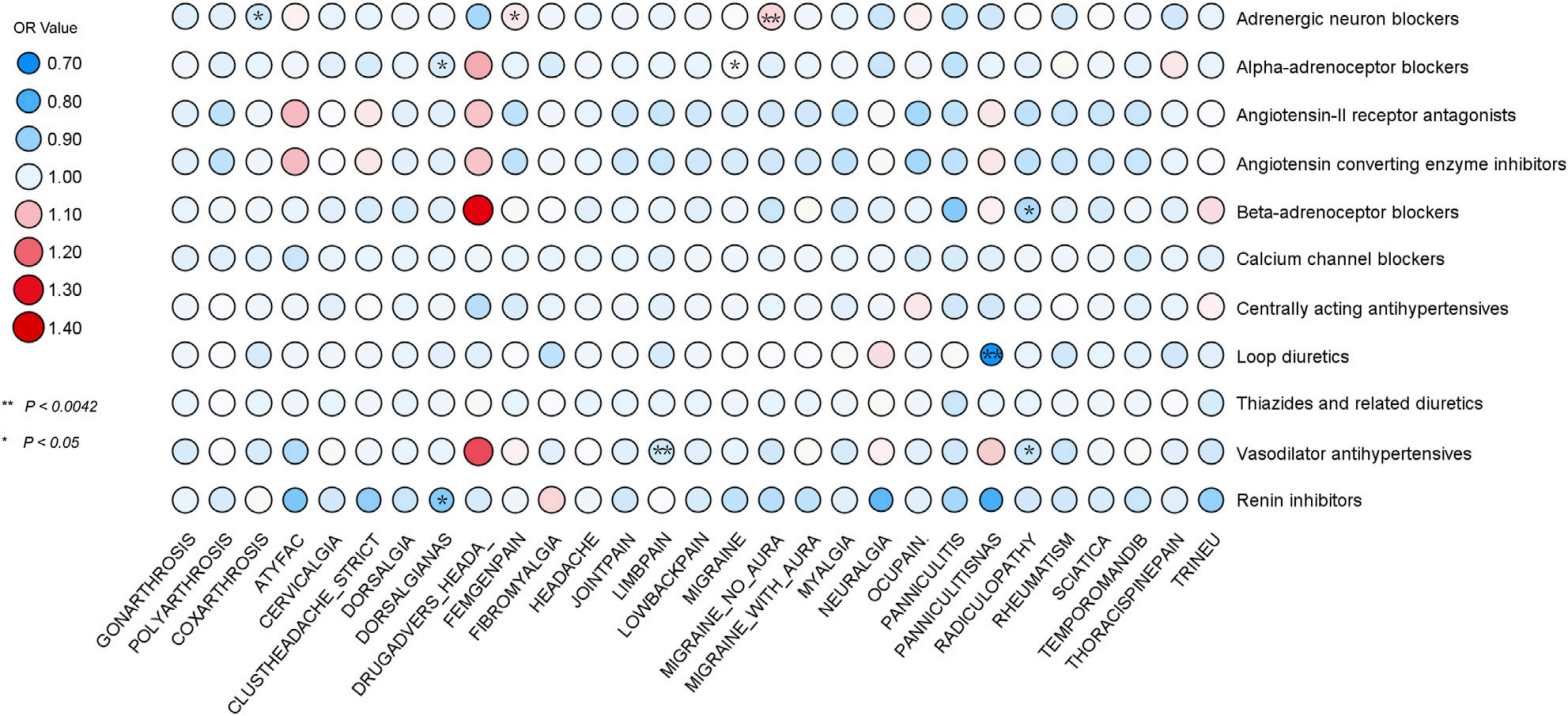
MR analysis using IVW identified associations between 11 antihypertensive drugs and 29 pain disorders. Associations with *p* < 0.0042 provided strong evidence, while 0.0042 < *p* < 0.05 indicated suggestive evidence.

In our comprehensive MR analysis, we evaluated the associations between 11 classes of antihypertensive drugs and 29 distinct pain disorders, with the detailed outcomes depicted in Figure 3. Notably, the analysis identified a significant association between adrenergic neuron blockers and an increased risk of migraine without aura, as evidenced by 12 SNPs [IVW: OR 1.07; 95% CI 1.028-1.118; *p* = 0.001]. Conversely, loop diuretics were associated with a significant protective effect against panniculitis, indicated by 11 SNPs [IVW: OR 0.73; 95% CI 0.591-0.890; *p* = 0.002], and vasodilator antihypertensives demonstrated a protective association against limb pain through 15 SNPs [IVW: OR 0.97; 95% CI 0.952-0.990; *p* = 0.003].

Sensitivity analyses including R^2^ and F-statistic values [Migraine without aura: R^2^ = 0.001, F = 52.478; panniculitis: R^2^ = 0.001, F = 67.129; Limb pain: R^2^ = 0.001, F = 51.001] underscored the instrumental variables’ strength and the analyses’ overall reliability. Furthermore, assessments of heterogeneity and pleiotropy revealed no significant concerns [Migraine without aura: Heterogeneity *p* = 0.078, Pleiotropy *p* = 0.587; panniculitis: Heterogeneity *p* = 0.390, Pleiotropy *p* = 0.508; Limb pain: Heterogeneity *p* = 0.193, Pleiotropy *p* = 0.850] confirming the stability of our findings (Supplementary Table S5). Steiger tests did not indicate reverse causation, affirming the directionality of their effects on pain disorders without evidence of reverse relationships. Supplementary Figures S1–S3 presents the diagrams for the sensitivity analysis, offering a visual representation of the robustness and reliability of our findings across various model specifications.

### 3.3 Suggestive evidence for the association between antihypertensive drugs and pain disorders

In our expanded analysis, where a more lenient *p*-value threshold was applied, we uncovered a wider array of associations between antihypertensive drugs and pain disorders, thereby enriching the robustness of our evidence base. Notably, adrenergic neuron blockers, analyzed using 10 SNPs, were associated with a decreased risk of coxarthrosis [IVW: OR = 0.97; 95% CI = 0.940-0.996; *p* = 0.027] and an increased risk for Femgenpain, a condition associated with pain and other disorders of the female genital organs and menstrual cycle, analyzed using 12 SNPs [IVW: OR = 1.05; 95% CI = 1.005-1.094; *p* = 0.027]. BBs, analyzed using 6 SNPs, showed a significant protective association with radiculopathy [IVW: OR = 0.93; 95% CI = 0.865-0.990; *p* = 0.024], a protective effect paralleled by vasodilator antihypertensives against radiculopathy, with 15 SNPs [IVW: OR = 0.96; 95% CI = 0.914-0.998; *p* = 0.044]. Alpha-adrenoceptor blockers demonstrated a protective effect against dorsalgianas, based on 15 SNPs [IVW: OR = 0.98; 95% CI = 0.950-0.999; *p* = 0.049], but an increased risk for migraine, analyzed with 14 SNPs [IVW: OR = 1.03; 95% CI = 1.004-1.057; *p* = 0.020]. To tackle the challenge of genetically proxied renin inhibitors with just a single SNP, we employed the Wald ratio model. This approach, crucial for its ability to offer precise causal estimates with minimal genetic data, demonstrated renin inhibitors’ significant protective effect against dorsalgianas [Wald ratio: OR = 0.88; 95% CI = 0.80-0.96; *p* = 0.004].

The F-statistic values, robust across the board [ranging from 46.931 to 62.659], alongside R^2^ metrics [spanning 0.0009 to 0.0005], underscore the instrumental variables’ strength and the analyses’ explanatory power. Although no significant heterogeneity was detected, pleiotropy was observed in the causal effect of adrenergic neuron blockers on coxarthrosis [Pleiotropy: *p* = 0.023]. Despite efforts to adjust for this through MR-PRESSO, leading to the removal of outlier SNPs, caution is warranted in interpreting these results due to a significant global test outcome [Global Test: *p* = 0.002]. Furthermore, Steiger testing revealed no evidence of reverse causality between antihypertensive drugs and pain disorders, thereby strengthening confidence in the associations’ directionality. Supplementary Figures S4–S9 feature forest, funnel, and leave-one-out plots, visually demonstrating the robustness of our findings by showcasing effect sizes, detecting potential bias, and verifying the consistency of the results across diverse analyses.

## 4 Discussion

Our study advances the understanding of antihypertensive drugs by highlighting their potential to impact pain management significantly, far exceeding their conventional role in blood pressure regulation. This exploration suggests a paradigm shift in treating individuals with concurrent hypertension and pain disorders, advocating for an integrated approach that capitalizes on the multifaceted pharmacological effects of these medications. By identifying robust associations between antihypertensive drugs and a range of pain conditions, we underscore the necessity of holistic treatment strategies that consider the complex relationship between cardiovascular health and pain modulation.

Migraine without aura involves intense headaches and sensory hypersensitivity, rooted in the trigeminovascular system’s dysregulation affecting brain’s vascular dynamics and inflammatory responses (Manzoni and Torelli, 2008; Waliszewska-Prosół et al., 2023). The sympathetic nervous system (SNS), which controls the body’s ‘fight or flight’ response, also plays a pivotal role in vascular regulation by controlling vasoconstriction and vasodilation (Schlereth and Birklein, 2008; Strong et al., 2020). Adrenergic neuron blockers, by inhibiting SNS activity, could inadvertently modulate migraine pathophysiology in several interconnected ways. Firstly, these blockers induce vasodilation by reducing SNS-driven vasoconstriction. This vasodilation, particularly in cerebral vessels, can trigger the early phase of a migraine attack, characterized by cortical spreading depression, neurogenic inflammation, and activation of pain pathways. The role of CGRP, a potent vasodilator released from sensory nerves, becomes crucial here (Russell et al., 2014). CGRP levels and activity are closely regulated by the SNS, specifically via α2 adrenergic receptors. Studies have demonstrated that the SNS can influence sensory CGRP systems, indicating a complex interplay where SNS activity modulates CGRP release and function (Kawasaki et al., 1990). This interaction suggests that adrenergic neuron blockers, by dampening SNS activity, might lead to an imbalance in CGRP levels, potentially enhancing its vasodilatory and pro-inflammatory effects in the context of migraines. Moreover, research involving models of hypertension has shown that interventions affecting CGRP levels can alter the sensory nerve activation without directly influencing blood pressure, underscoring CGRP’s role beyond vascular tone to include pain perception modulation (Fujioka et al., 1991). The suppression of NGF-stimulated CGRP release via α2 receptor-mediated pathways and the observed effects of α2 antagonists in restoring CGRP levels further illustrate the intricate relationship between SNS signaling, CGRP regulation, and hypertension-related processes, which may mirror similar mechanisms in migraine pathogenesis (Supowit et al., 1998).Therefore, the increased risk of migraine with aura associated with adrenergic neuron blockers might stem from these drugs’ broad systemic effects on the SNS and CGRP regulation. This includes altering cerebral blood flow through vasodilation and disrupting the balance of neuropeptides crucial for pain and inflammatory responses, such as CGRP. The findings point toward a need for further investigation into the SNS-CGRP axis as a target for migraine therapies, especially considering the protective and triggering roles of CGRP in migraine pathophysiology (Kawasaki et al., 1990). Our study identified discrepancies between our findings and clinical guidelines regarding the effects of adrenergic neuron blockers on migraine risk (Hovaguimian and Roth, 2022). Using a multivariable MR analysis that included caffeine intake, we confirmed an increased migraine risk associated with these blockers. Attempts at inverse MR were limited by insufficient instrumental variables. Despite this, our results suggest a complex interaction between genetic predispositions and drug effects, indicating the need for further research into the SNS-CGRP axis for potential migraine therapies.

Panniculitis, characterized by tender nodules and erythema within the subcutaneous fat layer, manifests from a variety of causes, ranging from infections to systemic diseases (Morrison et al., 2010). In contrast, loop diuretics, traditionally prescribed for controlling hypertension and fluid retention, act by inhibiting sodium and chloride reabsorption in the Loop of Henle within the kidneys (Malha and Mann, 2016). Our study introduces the speculative notion that loop diuretics might indirectly offer a novel therapeutic approach for managing panniculitis. By mitigating fluid retention, these medications could potentially alleviate the mechanical stress and inflammation in the subcutaneous fat layer, thereby providing symptomatic relief. Furthermore, the reduction in fluid overload might lead to a decrease in the body’s overall inflammatory state, indirectly benefiting conditions characterized by inflammation, such as panniculitis. Enhanced circulation, a secondary effect of the action of loop diuretics, could improve the removal of inflammatory mediators from affected tissues and support the healing processes within the subcutaneous fat layer impacted by panniculitis. Incorporating these speculative mechanisms into our understanding of panniculitis treatment suggests that loop diuretics could potentially serve as an adjunct therapy, particularly in cases where fluid retention exacerbates the condition. However, empirical research is needed to validate these hypotheses and to determine whether loop diuretics could indeed offer a beneficial effect on the management of panniculitis.

Vasodilator antihypertensives offer a multifaceted approach to improving vascular health and alleviating limb pain, underscoring the intrinsic connection between vascular function and pain perception. These medications enhance blood flow and reduce vascular resistance through several possible key mechanisms: enhancing nitric oxide availability for endothelial function improvement, directly inducing vasodilation, blocking endothelin receptors to counteract vasoconstriction, activating the prostacyclin pathway to improve microcirculation and inhibit thrombosis, and increasing perfusion pressure in compromised vascular territories. Such actions not only decrease peripheral resistance but also ensure a better blood supply to ischemic regions, directly addressing the pain associated with inadequate circulation. This improved blood flow is critical for relieving pain in conditions where ischemia or poor circulation is a contributing factor. By relaxing vascular smooth muscle and enhancing endothelial responses, these drugs mitigate the mechanical stress on blood vessels and support the delivery of oxygen and nutrients to affected tissues. The blockade of endothelin receptors and activation of prostacyclin pathways further contribute to vasodilation, offering additional routes to counteract the effects of vasoconstriction that exacerbates pain. The potential application of vasodilator antihypertensives in future treatment strategies for limb pain is promising, given their capacity to address the underlying vascular issues contributing to pain.

Our analysis also sheds light on suggestive evidence necessitating further investigation. Notably, the increased risk of femgenpain (Pain and other conditions associated with female genital organs and menstrual cycle) associated with adrenergic neuron blockers raises concerns about their use in conditions related to female reproductive health, suggesting a complex interaction with the SNS that warrants cautious application. Moreover, our observations on vasodilator antihypertensives suggest their utility in alleviating radiculopathy, further advocating for the exploration of their role in conditions characterized by nerve root irritation or compression. The tentative evidence surrounding alpha-adrenoceptor blockers and their dichotomous impact on conditions like dorsalgia and migraines, along with renin inhibitors’ potential protective effect against dorsalgianas, underscores the complex interplay between cardiovascular pharmacology and pain modulation. These findings prompt a comprehensive exploration into the cardiovascular system’s role in pain pathology and the therapeutic potential of antihypertensive drugs in pain management.

While previous studies have established BBs as effective in reducing perioperative pain and minimizing long-term opioid usage (Valdes et al., 2017; Nakafero et al., 2021), our findings introduce a novel perspective by identifying their protective role against radiculopathy. While previous research has touted the efficacy of BBs in mitigating OA symptoms, particularly in knee and hip joints, our study presents a divergent narrative. Our results do not support the effectiveness of BBs in OA management, instead highlighting the protective effects of adrenergic neuron blockers against coxarthrosis. By enhancing joint microcirculation and reducing inflammation, these drugs may present a novel strategy for managing OA pain, underscoring the need for targeted research to validate these findings.

Our research contributes to the complex discourse on the role of CCBs in pain management by revealing no significant association between CCBs and pain disorders. This finding contrasts sharply with previous studies suggesting their effectiveness in treating headaches (Tfelt-Hansen and Tfelt-Hansen, 2009). This discrepancy highlights the nuanced and multifaceted nature of pain modulation and the challenges inherent in repurposing CCBs for pain management. While biological rationales and their application in migraine treatment hint at a potential connection to pain conditions, the evidence from extensive human studies remains divided (Patel et al., 2018). Notably, some research points to worsened pain outcomes in conditions like knee OA (KOA) with CCB use, whereas other studies hint at potential analgesic properties (Li et al., 2021; Toikumo et al., 2023). These mixed outcomes underscore the necessity for a more discerning approach to employing CCBs in pain management, emphasizing the importance of further targeted research. Such studies are crucial for disentangling the beneficial from the potentially adverse effects of calcium channel modulation on various pain pathways.

Our exploration of antihypertensive drugs for pain management uncovers novel therapeutic possibilities and signifies a critical shift towards a personalized medicine approach for comorbid hypertension and pain. By tailoring treatments to individuals’ genetic profiles and the specific pharmacodynamics of these drugs, we anticipate substantial improvements in managing both conditions and enhancing patient outcomes. This direction necessitates further validation through targeted clinical trials, comprehensive longitudinal studies to assess long-term effects, and fundamental scientific research to elucidate molecular mechanisms, ensuring that our findings effectively inform and refine clinical guidelines.

While our study employs a robust MR framework to investigate the role of antihypertensive drugs in pain management, the reliance on genetic data predominantly from populations of European ancestry limits the generalizability of our findings. To address this limitation, future research must incorporate GWAS from multi-ethnic cohorts. Expanding the diversity of the study population will reduce potential biases and improve the representativeness of our results across diverse global populations, thereby supporting the universal applicability of our conclusions.

Additionally, the effectiveness of our MR approach is constrained by the current scope of genetic knowledge, which may overlook critical genetic variants and interactions essential for a comprehensive understanding of drug efficacy. To mitigate this limitation, we will integrate newly identified genetic data from ongoing studies and continuously update our genetic instruments with emerging variants. This step will broaden the spectrum of genetic influences analyzed, ensuring that our results are robust and relevant for personalized medicine across various demographics.

Our use of summary-level data in MR analysis, while practical, limits the granularity of our findings and may overlook critical genetic variants and interactions. To address these limitations, future research should incorporate individual-level data where feasible. This approach will provide a more detailed understanding of genetic associations and their implications for pain management. Additionally, addressing potential biases due to population stratification and unmeasured confounders is crucial. Implementing advanced statistical methods and collaborating with international biobanks can enhance the robustness and applicability of our results.

The complexity of the pharmacological effects of antihypertensive drugs on pain disorders underscores the necessity of interdisciplinary collaboration. By merging expertise from pharmacology, neurology, and pain medicine, we can develop integrated treatment strategies that offer innovative and effective therapies. This collaborative effort is crucial for advancing our understanding of the interconnections between cardiovascular health and pain, leading to more nuanced treatment options and significant progress in both hypertension management and pain medicine.

Translating our findings into clinical practice requires rigorous validation. Future clinical trials must assess the efficacy and safety of repurposing antihypertensive drugs for pain management, particularly in chronic pain patients with comorbid hypertension. These trials should evaluate the drugs in various clinical settings, focusing on key metrics such as pain reduction, quality of life improvements, and potential adverse effects. Subsequent longitudinal studies will be necessary to assess long-term outcomes and safety. This systematic approach will ensure that our research is both clinically relevant and applicable, supporting precision medicine initiatives.

In conclusion, our study highlights the potential of repurposing antihypertensive drugs for pain management and underscores the importance of a cohesive, evidence-based approach. Addressing the identified limitations through specific methodological enhancements, broader data inclusion, and detailed clinical translation plans will significantly increase the impact and relevance of our findings. A comprehensive and nuanced discussion of these aspects is essential to fully realize the potential of our research and ensure effective translation into clinical practice.

## 5 Conclusion

Our study delves into the repurposing of antihypertensive drugs for pain management, uncovering both beneficial and potential adverse effects on various pain disorders through the MR approach. Adrenergic neuron blockers show promise in protecting against coxarthrosis. Still, they might increase the risk of femgenpain and migraine without aura, indicating a need for careful consideration in their use for pain management. Similarly, while alpha-adrenoceptor blockers are linked to a higher risk of migraine, they, along with renin inhibitors, offer a protective effect against dorsalgianas. Furthermore, our findings suggest that BBs and vasodilator antihypertensives reduce the risk of radiculopathy, with the latter also beneficial for limb pain, and loop diuretics showing a protective impact on panniculitis.

## 6 Limitations

Our findings are based on genetic data that predominantly represents European ancestries and relies on existing genetic knowledge, potentially limiting their applicability across diverse global populations. This reliance may overlook critical genetic variants and interactions, affecting the study’s outcomes and the generalizability of our results. Additionally, our study is constrained using summary-level data, which limits the granularity of our findings. The potential for biases due to population stratification and the predominance of European ancestry in the genetic data further restricts the applicability of our conclusions to other populations. Despite rigorous analytical tests, the potential for pleiotropy—where genetic variants influence multiple traits—could introduce confounding effects, highlighting the complexity of genetic associations within our study context. Moreover, translating our findings into clinical practice faces significant challenges. The need for clinical trials to confirm the safety and efficacy of these drugs for pain management underscores that our findings are preliminary steps toward clinical application. Furthermore, the methodological constraints of MR analysis and the focus on broad drug classes and specific pain conditions may oversimplify the intricate interactions between drugs and pain disorders.

## Data Availability

All data used in this study were obtained from IEU Open GWAS (https://gwas.mrcieu.ac.uk/).
