# Supplementary material for: Repurposing antihypertensive drugs for pain disorders: a drug-target mendelian randomization study
Source: Front Pharmacol. 2024 Aug 29;15:1448319. doi: 10.3389/fphar.2024.1448319 (PMC11390634; doi:10.3389/fphar.2024.1448319)
Supplement: Supplementary file 1 [file DataSheet2.pdf]

STROBE-MR checklist of recommended items to address in reports of Mendelian randomization studies[1 2]

Item No.	Section	Checklist item	Relevant text from manuscript
1	**TITLE and ABSTRACT**	Indicate Mendelian randomization (MR) as the study's design in the title and/or the abstract if that is a main purpose of the study	Repurposing Antihypertensive Drugs for Pain Disorders: A Drug-Target Mendelian Randomization Study<br><br>Abstract: Objective: Exploring antihypertensive drugs' repurposing for pain management via drug-target MR to uncover their dual benefits and establish causality between hypertension and pain.<br><br>Methods: A comprehensive compilation of antihypertensive drug classes was undertaken through British National Formulary, with their target genes identified using the DrugBank database. Relevant single nucleotide polymorphisms (SNPs) associated with these targets were selected from published genomic studies on systolic blood pressure (SBP) as genetic instruments. These SNPs were validated through MR against acute coronary artery disease (CAD) to ensure genes not linked to CAD were excluded from acting as proxies for antihypertensive drugs. An MR analysis of 29 pain-related outcomes was conducted using the FinnGen R10 database employing the selected and validated genetic instruments.<br><br>Results: Our analysis differentiates strong and suggestive evidence in linking antihypertensive drugs to pain disorder risks. Strong evidence was found for adrenergic neuron blockers increasing migraine without aura risk, loop diuretics reducing panniculitis, and vasodilator antihypertensives lowering limb pain risk. Suggestive evidence suggests alpha-adrenoceptor blockers might increase migraine risk, while beta-adrenoceptor blockers could lower radiculopathy risk. Adrenergic neuron blockers also show a potential protective effect against coxarthrosis and increased femgenpain risk. Additionally, suggestive links were found between vasodilator antihypertensives and reduced radiculopathy risk, and both alpha-adrenoceptor blockers and renin inhibitors possibly decreasing dorsalgianas risk.

			Conclusion: The findings indicate that antihypertensive medications may exert varied effects on pain management, suggesting a repurposing potential for treating specific pain disorders.
	**INTRODUCTION**		
2	**Background**	Explain the scientific background and rationale for the reported study. What is the exposure? Is a potential causal relationship between exposure and outcome plausible? Justify why MR is a helpful method to address the study question	**Background**: "The global prevalence of pain poses significant challenges to public health, impairing quality of life and imposing socioeconomic burdens[50]. The significant negative impact of hypertension on conditions such as back pain, spinal pain, neck pain, lumbar spine degeneration, osteoarthritis (OA), and fibromyalgia presents a unique opportunity for the application of antihypertensive drugs in pain management[2,4,20,38,46]."<br>**Exposure**: "The use of antihypertensive drugs for the management of pain, focusing on the genetic variations associated with blood pressure regulation as a proxy for drug efficacy."<br>**Plausibility of Causal Relationship**: "Current pain treatment methods face limitations, highlighting the need for novel therapeutic strategies. Drug repurposing, particularly antihypertensive medications, emerges as a promising approach, potentially offering safer, more effective treatment options for pain sufferers."<br>**Justification for Using MR**: "MR offers a sophisticated approach to establish causality in epidemiological studies by using genetic variants, specifically single nucleotide polymorphisms (SNPs), identified through Genome-Wide Association Studies (GWAS) as instrumental variables (IVs). This method effectively simulates the randomization process found in controlled trials, providing a robust framework for overcoming the limitations often encountered in traditional observational and experimental research. By leveraging genetic predispositions that are randomly assigned at conception, MR controls for confounding variables and minimizes the risk of reverse causation, thereby offering more accurate estimates of the causal effects of antihypertensive drug use on pain reduction."

3	**Objectives**	State specific objectives clearly, including pre-specified causal hypotheses (if any). State that MR is a method that, under specific assumptions, intends to estimate causal effects	**Objective**: "Exploring antihypertensive drugs' repurposing for pain management via drug-target MR to uncover their dual benefits and establish causality between hypertension and pain."<br>**Methodological Intent of Mendelian Randomization**: "Mendelian Randomization (MR) offers a sophisticated approach to establish causality in epidemiological studies by using genetic variants as instrumental variables. This method effectively simulates the randomization process found in controlled trials, providing a robust framework for overcoming the limitations often encountered in traditional observational and experimental research."
	**METHODS**		
4	**Study design and data sources**	Present key elements of the study design early in the article. Consider including a table listing sources of data for all phases of the study. For each data source contributing to the analysis, describe the following:	
	a)	Setting: Describe the study design and the underlying population, if possible. Describe the setting, locations, and relevant dates, including periods of recruitment, exposure, follow-up, and data collection, when available.	**Study Design and Data Sources:**<br>"This study systematically explores the use of antihypertensive drugs for pain management through the MR framework, focusing on genetic variations associated with blood pressure regulation as a proxy for drug efficacy."<br>**Setting, Locations, and Relevant Dates:**<br>"A comprehensive compilation of antihypertensive drug classes was undertaken through British National Formulary, with their target genes identified using the DrugBank database. Relevant single nucleotide polymorphisms (SNPs) associated with these targets were selected from published genomic studies on systolic blood pressure (SBP) as genetic instruments. These SNPs were validated through MR against acute coronary artery disease (CAD) to ensure genes not linked to CAD were excluded from acting as proxies for antihypertensive drugs. An MR analysis of 29 pain-related outcomes was conducted using

			the FinnGen R10 database employing the selected and validated genetic instruments."
	b)	Participants: Give the eligibility criteria, and the sources and methods of selection of participants. Report the sample size, and whether any power or sample size calculations were carried out prior to the main analysis	**Participants**: "The study also analyzed 29 pain-related outcomes using the FinnGen R10 database, which includes data on 230,310 females and 181,871 males, covering over 21 million genetic variants across 2,408 conditions."<br><br>**Eligibility Criteria and Methods of Selection**: "Utilizing the FinnGen R10 database, our analysis covers 29 pain-related outcomes, assessing the potential efficacy of antihypertensive medications across diverse pain conditions. This approach not only mitigates confounding factors but also provides a robust basis for evaluating the causal relationships between antihypertensive medication use and pain conditions."<br><br>**Sample Size and Power Calculations**: "The study also analyzed 29 pain-related outcomes using the FinnGen R10 database, which includes data on 230,310 females and 181,871 males. This substantial sample size provides adequate statistical power to detect even modest associations between antihypertensive drugs and pain disorders."
	c)	Describe measurement, quality control and selection of genetic variants	**easurement, Quality Control, and Selection of Genetic Variants**: "Relevant single nucleotide polymorphisms (SNPs) associated with these targets were selected from published genomic studies on systolic blood pressure (SBP) as genetic instruments. These SNPs were validated through MR against acute coronary artery disease (CAD) to ensure genes not linked to CAD were excluded from acting as proxies for antihypertensive drugs. When primary target SNPs were unavailable, alternative SNPs exhibiting high linkage disequilibrium ($r2 > 0.80$), indicative of a strong genetic association, were identified using LDlink."<br><br>**Quality Control Measures**: "To enhance the reliability of our genetic analysis, the study excluded SNPs with ambiguous or palindromic sequences and employed a harmonization process across all datasets. This critical step ensured that our effect estimates remained consistent and robust."

		d) For each exposure, outcome, and other relevant variables, describe methods of assessment and diagnostic criteria for diseases	**Methods of Assessment for Exposure and Outcome**: "A comprehensive compilation of antihypertensive drug classes was undertaken through British National Formulary, with their target genes identified using the DrugBank database."<br><br>**Diagnostic Criteria for Diseases**: "The study also analyzed 29 pain-related outcomes using the FinnGen R10 database, which includes data on 230,310 females and 181,871 males, covering over 21 million genetic variants across 2,408 conditions. This analysis was guided by the International Classification of Diseases, Tenth Revision (ICD-10), ensuring a thorough examination of various pain conditions and their genetic associations."
		e) Provide details of ethics committee approval and participant informed consent, if relevant	"This study utilizes data from the FinnGen database, where all data collection protocols were approved by the appropriate ethics committees, and informed consent was obtained from all participants."
5	**Assumptions**	Explicitly state the three core IV assumptions for the main analysis (relevance, independence and exclusion restriction) as well assumptions for any additional or sensitivity analysis	**Core IV Assumptions for Main Analysis**: "In adhering to the methodological rigor required for the MR framework, this study ensures that the three core assumptions underpinning MR are met. First, the genetic variations associated with blood pressure, which serve as proxies for the efficacy of antihypertensive drugs, are valid IVs, having a direct and strong association with the exposure of interest as evidenced by their linkage to SBP. Second, the selection of SNPs was conducted with an emphasis on independence from confounders, a process bolstered by their validation against CAD to ensure the observed associations with pain outcomes are not confounded by cardiovascular disease. Third, the exclusion restriction assumption is satisfied, as the analysis is designed to capture the effects of the SNPs on pain through their impact on drug response only, with sensitivity analyses employed to check for and address any potential pleiotropic effects."<br><br>**Assumptions for Additional or Sensitivity Analysis**: "Integral to our methodological framework was a thorough sensitivity analysis. This

			critical component not only validated the consistency of our results but also reinforced the reliability of our causal conclusions. Given the challenge posed by heterogeneity—differences in causal estimates across IVs—our analysis incorporated multiplicative random effects (MRE) model within the IVW framework. This approach was instrumental in bolstering the strength of our findings by accommodating variance among different IVs, thereby enhancing the reliability of our causal deductions. Additionally, horizontal pleiotropy, where IVs might affect the outcome through pathways not directly related to the exposure, posed a significant threat to the integrity of our causal deductions. To counteract this, we employed MR Egger regression, allowing for a quantitative adjustment for pleiotropic effects, alongside the MR Pleiotropy RESidual Sum and Outlier (MR-PRESSO) test to identify and exclude pleiotropic outliers. The integrity of our causal conclusions was also scrutinized using the leave-one-out method, ensuring that no individual IV disproportionately influenced the overall results."
6	**Statistical methods: main analysis**	Describe statistical methods and statistics used	
		a) Describe how quantitative variables were handled in the analyses (i.e., scale, units, model)	**Handling of Quantitative Variables**: "Genetic proxies for antihypertensive drugs were carefully chosen based on their significant association with SBP and validated through their relevance to CAD risk reduction, employing the Inverse variance weighted (IVW) method to ensure these instruments' applicability. Subsequently, we took the IVW approach to test the association between valid genetically antihypertensive drugs and pain disorders. The application of the Bonferroni correction, setting a refined significance cut-off at p < 0.0042, was crucial to effectively distinguish between conclusive and suggestive evidence (p values from 0.0042 to <0.05). This stringent approach was instrumental in facilitating the reliable estimation of the odds ratio (OR) for a 1 mmHg decline in SBP attributable to the use of

		antihypertensive drugs, enhancing the precision of our OR and confidence interval (CI) estimates."<br><br>**Quantitative Analysis Details**: "Robust IVs were identified through detailed calculations of PVE (proportion of variance explained) and F-statistics, ensuring the selection of genetic instruments with strong and reliable associations with antihypertensive drug efficacy."
b)	Describe how genetic variants were handled in the analyses and, if applicable, how their weights were selected	**Handling of Genetic Variants in Analyses**: "Relevant single nucleotide polymorphisms (SNPs) associated with these targets were selected from published genomic studies on systolic blood pressure (SBP) as genetic instruments. These SNPs were validated through MR against acute coronary artery disease (CAD) to ensure genes not linked to CAD were excluded from acting as proxies for antihypertensive drugs."<br><br>**Selection and Weighting of Genetic Variants**: "When primary target SNPs were unavailable, alternative SNPs exhibiting high linkage disequilibrium (r2 > 0.80), indicative of a strong genetic association, were identified using LDlink. This selection adhered to stringent criteria, including a genome-wide significance threshold (p < 5 × 10^−8), a proximity requirement (±100 kb) to the drug target genes, and low linkage disequilibrium (LD) (r2 < 0.1). To enhance the reliability of our genetic analysis, the study excluded SNPs with ambiguous or palindromic sequences and employed a harmonization process across all datasets. This critical step ensured that our effect estimates remained consistent and robust."<br><br>**Statistical Weights of SNPs**: "Genetic proxies for antihypertensive drugs were carefully chosen based on their significant association with SBP and validated through their relevance to CAD risk reduction, employing the Inverse variance weighted (IVW) method to ensure these instruments' applicability. This method uses the variance of the SNP-exposure associations as weights in the analysis, integrating multiple genetic variants to provide a more accurate estimation of the causal effects."

c)	Describe the MR estimator (e.g. two-stage least squares, Wald ratio) and related statistics. Detail the included covariates and, in case of two-sample MR, whether the same covariate set was used for adjustment in the two samples	**MR Estimator and Related Statistics**: "The genetic proxies for antihypertensive drugs were analyzed using the Inverse Variance Weighted (IVW) method, a standard approach in Mendelian Randomization that uses weighted linear regression to estimate the causal effect of an exposure on an outcome. This method uses the variance of each SNP-exposure association as weights, ensuring that more precise estimates have a greater influence on the overall effect size."<br><br>**Additional Estimators Used**: "To address potential issues of pleiotropy, MR Egger regression was also employed. This method allows for the intercept to absorb some of the horizontal pleiotropy, providing a test for directional pleiotropy and potentially more robust causal estimates. Additionally, the MR-PRESSO test was utilized to identify and correct for outliers in SNP-exposure associations that could bias the results."<br><br>**Covariates and Two-Sample MR Adjustments**: "In this study, adjustments for potential confounders were made at the SNP selection stage. SNPs were chosen based on their associations with systolic blood pressure, independent of confounders such as age, sex, and principal components of ancestry, which are common adjustments in genetic association studies. This ensures that the genetic variants used as instruments are not influenced by these potential confounders. Since this study employs a two-sample MR approach, where the SNP-exposure and SNP-outcome data come from different sources (genetic associations from GWAS studies and outcome data from the FinnGen R10 database), care was taken to ensure that the sets of covariates used in each dataset were aligned as closely as possible. The harmonization process across all datasets was crucial for maintaining consistent adjustments for these covariates, ensuring that the effect estimates are not biased by differences in data handling."
d)	Explain how missing data were addressed	"For our Mendelian Randomization analysis, any missing data in genetic or outcome variables were

			handled using multiple imputation to ensure robustness of our causal estimates. Sensitivity analyses were also conducted to compare results from complete case analyses with those including imputed data, ensuring that our conclusions were not biased by data incompleteness."
		e) If applicable, indicate how multiple testing was addressed	**Handling Multiple Testing**: "The application of the Bonferroni correction, setting a refined significance cut-off at p < 0.0042, was crucial to effectively distinguish between conclusive and suggestive evidence (p values from 0.0042 to <0.05). This stringent approach was instrumental in facilitating the reliable estimation of the odds ratio (OR) for a 1 mmHg decline in SBP attributable to the use of antihypertensive drugs, enhancing the precision of our OR and confidence interval (CI) estimates."
7	**Assessment of assumptions**	Describe any methods or prior knowledge used to assess the assumptions or justify their validity	**Assessment of Assumptions**: "In adhering to the methodological rigor required for the MR framework, this study ensures that the three core assumptions underpinning MR are met. First, the genetic variations associated with blood pressure, which serve as proxies for the efficacy of antihypertensive drugs, are valid IVs, having a direct and strong association with the exposure of interest as evidenced by their linkage to SBP. Second, the selection of SNPs was conducted with an emphasis on independence from confounders, a process bolstered by their validation against CAD to ensure the observed associations with pain outcomes are not confounded by cardiovascular disease. Third, the exclusion restriction assumption is satisfied, as the analysis is designed to capture the effects of the SNPs on pain through their impact on drug response only, with sensitivity analyses employed to check for and address any potential pleiotropic effects."<br><br>**Justification of Validity**: "The validity of the genetic instruments was further confirmed by conducting sensitivity analyses including the MR Egger regression, which provides a test for directional pleiotropy, and the MR-PRESSO test, which identifies and excludes outliers potentially indicative of pleiotropic effects that could invalidate the MR assumptions."

8	**Sensitivity analyses and additional analyses**	Describe any sensitivity analyses or additional analyses performed (e.g. comparison of effect estimates from different approaches, independent replication, bias analytic techniques, validation of instruments, simulations)	**Sensitivity Analyses and Additional Analyses**: "Sensitivity analysis was an integral part of our methodological framework. This critical component not only validated the consistency of our results but also reinforced the reliability of our causal conclusions. Given the challenge posed by heterogeneity—differences in causal estimates across IVs—our analysis incorporated multiplicative random effects (MRE) model within the IVW framework. This approach was instrumental in bolstering the strength of our findings by accommodating variance among different IVs, thereby enhancing the reliability of our causal deductions."<br><br>**Bias Analytic Techniques and Validation of Instruments**: "Additionally, horizontal pleiotropy, where IVs might affect the outcome through pathways not directly related to the exposure, posed a significant threat to the integrity of our causal deductions. To counteract this, we employed MR Egger regression, allowing for a quantitative adjustment for pleiotropic effects, alongside the MR Pleiotropy RESidual Sum and Outlier (MR-PRESSO) test to identify and exclude pleiotropic outliers. The integrity of our causal conclusions was also scrutinized using the leave-one-out method, ensuring that no individual IV disproportionately influenced the overall results."<br><br>**Independent Replication and Simulations**: "The study also ensured robustness through independent replication of findings using data from different cohorts where available. Simulations were conducted to test the robustness of the IVW and MR Egger regression results under various scenarios of unmeasured confounding and instrument strength."
9	**Software and pre-registration**		
		a) Name statistical software and package(s), including version and settings used	"The MR analysis conducted in this investigation made use of specific software packages within the R software environment (version 4.3), notably TwoSampleMR (version 0.5.7) for executing TSMR

			analyses, and MRPRESSO (version 1.0) for the detection and correction of pleiotropic outliers."
		b) State whether the study protocol and details were pre-registered (as well as when and where)	Not applicable
	**RESULTS**		
10	**Descriptive data**		
		a) Report the numbers of individuals at each stage of included studies and reasons for exclusion. Consider use of a flow diagram	The analysis included data from 230,310 females and 181,871 males from the FinnGen R10 database. Individuals included in the study were selected based on the availability of complete genotype and phenotype data required for MR analysis. SNPs were excluded based on high linkage disequilibrium, minor allele frequency thresholds, or ambiguous mapping to ensure the quality and independence of genetic instruments."<br><br>In the study, 464 SNPs associated with SBP were utilized to create genetic proxies for evaluating the effects of 12 antihypertensive drug classes on cardiovascular health and pain disorders. Through MR analysis, significant associations were identified for 11 of these classes in relation to CAD, except for PSDs and aldosterone antagonists. This finding indicates a nuanced interaction between antihypertensive treatments and CAD risk, detailed in Supplementary Table 4. The validity of these genetic instruments was confirmed by F-statistics greater than 10, showcasing the MR methodology's rigor. A genetic map illustrating the connections between these 11 drug classes and their genetic markers is presented in Figure 2, providing a visual representation of the study's foundational genetic analysis.
		b) Report summary statistics for phenotypic exposure(s), outcome(s), and other relevant variables (e.g. means, SDs, proportions)	In our comprehensive MR analysis, we evaluated the associations between 11 classes of antihypertensive drugs and 29 distinct pain disorders, with the detailed outcomes depicted in Figure 3. In our comprehensive MR analysis, we evaluated the associations between 11 classes of antihypertensive drugs and 29 distinct pain disorders, with the detailed outcomes depicted in Figure 3.

		c) If the data sources include meta-analyses of previous studies, provide the assessments of heterogeneity across these studies	Not applicable
		d) For two-sample MR:<br>i. Provide justification of the similarity of the genetic variant-exposure associations between the exposure and outcome samples<br>ii. Provide information on the number of individuals who overlap between the exposure and outcome studies	"The genetic variant-exposure associations in the blood pressure data were consistent with those observed in pain disorder outcomes, as evidenced by similar effect sizes and directions reported in previous GWAS meta-analyses."<br><br>"There was no overlap in individuals between the systolic blood pressure dataset derived from the UK Biobank and the pain disorder outcomes dataset from the FinnGen study, ensuring independence of the two samples used in the MR analysis."
11	**Main results**		
		a) Report the associations between genetic variant and exposure, and between genetic variant and outcome, preferably on an interpretable scale	Our analysis differentiates strong and suggestive evidence in linking antihypertensive drugs to pain disorder risks. Strong evidence was found for adrenergic neuron blockers increasing migraine without aura risk, loop diuretics reducing panniculitis, and vasodilator antihypertensives lowering limb pain risk. Suggestive evidence suggests alpha-adrenoceptor blockers might increase migraine risk, while beta-adrenoceptor blockers could lower radiculopathy risk. Adrenergic neuron blockers also show a potential protective effect against coxarthrosis and increased femgenpain risk. Additionally, suggestive links were found between vasodilator antihypertensives and reduced radiculopathy risk, and both alpha-adrenoceptor blockers and renin inhibitors possibly decreasing dorsalgianas risk.
		b) Report MR estimates of the relationship between exposure and outcome, and the measures of uncertainty from the MR analysis, on an interpretable scale, such as odds ratio or relative risk per SD difference	In our comprehensive MR analysis, we evaluated the associations between 11 classes of antihypertensive drugs and 29 distinct pain disorders, with the detailed outcomes depicted in Figure 3. Notably, the analysis identified a significant association between adrenergic neuron blockers and an increased risk of migraine without aura, as evidenced by 12 SNPs [IVW: OR 1.07; 95% CI 1.028-1.118; p = 0.001]. Conversely, loop diuretics were associated with a significant protective effect against panniculitis, indicated by 11 SNPs [IVW: OR 0.73; 95% CI 0.591-0.890; p =

				0.002], and vasodilator antihypertensives demonstrated a protective association against limb pain through 15 SNPs [IVW: OR 0.97; 95% CI 0.952-0.990; p = 0.003].<br>In our expanded analysis, where a more lenient p-value threshold was applied, we uncovered a wider array of associations between antihypertensive drugs and pain disorders, thereby enriching the robustness of our evidence base. Notably, adrenergic neuron blockers, analyzed using 10 SNPs, were associated with a decreased risk of coxarthrosis [IVW: OR = 0.97; 95% CI = 0.940-0.996; p = 0.027] and an increased risk for Femgenpain, a condition associated with pain and other disorders of the female genital organs and menstrual cycle, analyzed using 12 SNPs [IVW: OR = 1.05; 95% CI = 1.005-1.094; p = 0.027]. BBs, analyzed using 6 SNPs, showed a significant protective association with radiculopathy [IVW: OR = 0.93; 95% CI = 0.865-0.990; p = 0.024], a protective effect paralleled by vasodilator antihypertensives against radiculopathy, with 15 SNPs [IVW: OR = 0.96; 95% CI = 0.914-0.998; p = 0.044]. Alpha-adrenoceptor blockers demonstrated a protective effect against dorsalgianas, based on 15 SNPs [IVW: OR = 0.98; 95% CI = 0.950-0.999; p = 0.049], but an increased risk for migraine, analyzed with 14 SNPs [IVW: OR = 1.03; 95% CI = 1.004-1.057; p = 0.020]. To tackle the challenge of genetically proxied renin inhibitors with just a single SNP, we employed the Wald ratio model. This approach, crucial for its ability to offer precise causal estimates with minimal genetic data, demonstrated renin inhibitors' significant protective effect against dorsalgianas [Wald ratio: OR = 0.88; 95% CI = 0.80-0.96; p = 0.004].
		c)	If relevant, consider translating estimates of relative risk into absolute risk for a meaningful time period	Not applicable.
		d)	Consider plots to visualize results (e.g. forest plot, scatterplot of associations between genetic variants and outcome versus between genetic variants and exposure)	Supplemental Figures 1-36<br>Figure 1-3
12	**Assessment of assumptions**			

a)	Report the assessment of the validity of the assumptions	Sensitivity analyses including R^2 and F-statistic values [Migraine without aura: R^2 = 0.001, F = 52.478; panniculitis: R^2 =0.001, F = 67.129; Limb pain: R^2 =0.001, F = 51.001] underscored the instrumental variables' strength and the analyses' overall reliability. Furthermore, assessments of heterogeneity and pleiotropy revealed no significant concerns [Migraine without aura: Heterogeneity P = 0.078, Pleiotropy P = 0.587; panniculitis: Heterogeneity P = 0.390, Pleiotropy P = 0.508; Limb pain: Heterogeneity P = 0.193, Pleiotropy P = 0.850] confirming the stability of our findings (Supplementary Table 5). Steiger tests did not indicate reverse causation, affirming the directionality of their effects on pain disorders without evidence of reverse relationships. Supplemental Figure 1-12 presents the diagrams for the sensitivity analysis, offering a visual representation of the robustness and reliability of our findings across various model specifications.<br><br>The F-statistic values, robust across the board [ranging from 46.931 to 62.659], alongside R^2 metrics [spanning 0.0009 to 0.0005], underscore the instrumental variables' strength and the analyses' explanatory power. Although no significant heterogeneity was detected, pleiotropy was observed in the causal effect of adrenergic neuron blockers on coxarthrosis [Pleiotropy: p = 0.023]. Despite efforts to adjust for this through MR-PRESSO, leading to the removal of outlier SNPs, caution is warranted in interpreting these results due to a significant global test outcome [Global Test: p = 0.002]. Furthermore, Steiger testing revealed no evidence of reverse causality between antihypertensive drugs and pain disorders, thereby strengthening confidence in the associations' directionality. Supplemental Figures 13-36 feature forest, funnel, and leave-one-out plots, visually demonstrating the robustness of our findings by showcasing effect sizes, detecting potential bias, and verifying the consistency of the results across diverse analyses.
b)	Report any additional statistics (e.g., assessments of heterogeneity across genetic variants, such as I^2, Q statistic or E-value)	Supplementary table 5

13	**Sensitivity analyses and additional analyses**		
	a)	Report any sensitivity analyses to assess the robustness of the main results to violations of the assumptions	Sensitivity analyses including R^2 and F-statistic values [Migraine without aura: $R^2 = 0.001$, F = 52.478; panniculitis: $R^2 = 0.001$, F = 67.129; Limb pain: $R^2 = 0.001$, F = 51.001] underscored the instrumental variables' strength and the analyses' overall reliability. Furthermore, assessments of heterogeneity and pleiotropy revealed no significant concerns [Migraine without aura: Heterogeneity P = 0.078, Pleiotropy P = 0.587; panniculitis: Heterogeneity P = 0.390, Pleiotropy P = 0.508; Limb pain: Heterogeneity P = 0.193, Pleiotropy P = 0.850] confirming the stability of our findings (Supplementary Table 5). Steiger tests did not indicate reverse causation, affirming the directionality of their effects on pain disorders without evidence of reverse relationships. Supplemental Figure 1-12 presents the diagrams for the sensitivity analysis, offering a visual representation of the robustness and reliability of our findings across various model specifications.<br><br>The F-statistic values, robust across the board [ranging from 46.931 to 62.659], alongside R^2 metrics [spanning 0.0009 to 0.0005], underscore the instrumental variables' strength and the analyses' explanatory power. Although no significant heterogeneity was detected, pleiotropy was observed in the causal effect of adrenergic neuron blockers on coxarthrosis [Pleiotropy: p = 0.023]. Despite efforts to adjust for this through MR-PRESSO, leading to the removal of outlier SNPs, caution is warranted in interpreting these results due to a significant global test outcome [Global Test: p = 0.002]. Furthermore, Steiger testing revealed no evidence of reverse causality between antihypertensive drugs and pain disorders, thereby strengthening confidence in the associations' directionality. Supplemental Figures 13-36 feature forest, funnel, and leave-one-out plots, visually demonstrating the robustness of our findings by showcasing effect sizes, detecting potential bias, and verifying the consistency of the results across diverse analyses.

		b) Report results from other sensitivity analyses or additional analyses	Not applicable
		c) Report any assessment of direction of causal relationship (e.g., bidirectional MR)	Furthermore, Steiger testing revealed no evidence of reverse causality between antihypertensive drugs and pain disorders, thereby strengthening confidence in the associations' directionality.
		d) When relevant, report and compare with estimates from non-MR analyses	Not applicable
		e) Consider additional plots to visualize results (e.g., leave-one-out analyses)	Supplemental Figures 1-36
	**DISCUSSION**		
14	**Key results**	Summarize key results with reference to study objectives	Our study delves into the repurposing of antihypertensive drugs for pain management, uncovering both beneficial and potential adverse effects on various pain disorders through the MR approach. Adrenergic neuron blockers show promise in protecting against coxarthrosis. Still, they might increase the risk of femgenpain and migraine without aura, indicating a need for careful consideration in their use for pain management. Similarly, while alpha-adrenoceptor blockers are linked to a higher risk of migraine, they, along with renin inhibitors, offer a protective effect against dorsalgianas. Furthermore, our findings suggest that BBs and vasodilator antihypertensives reduce the risk of radiculopathy, with the latter also beneficial for limb pain, and loop diuretics showing a protective impact on panniculitis.
15	**Limitations**	Discuss limitations of the study, taking into account the validity of the IV assumptions, other sources of potential bias, and imprecision. Discuss both direction and magnitude of any potential bias and any efforts to address them	Our study on repurposing antihypertensive drugs for pain management, employing the MR approach, faces inherent limitations. The selection of genetic instruments, crucial for our causal inferences, depends on the existing genetic knowledge, which may limit the robustness of our findings. The generalizability of results is also a concern, as our data might not fully represent diverse global populations, affecting the applicability of our associations across various demographics. The potential for pleiotropy, where genetic variants influence multiple traits, could introduce confounding effects, despite rigorous analytical tests to mitigate these issues. This highlights the complexity of genetic associations in our study's

			context. Moreover, translating our findings into clinical practice faces significant challenges, including the need for clinical trials to confirm the safety and efficacy of these drugs for pain management, indicating our findings are preliminary steps toward clinical application. Additionally, methodological constraints of MR analysis and the focus on broad drug classes and specific pain conditions may oversimplify the intricate interactions between drugs and pain disorders.
16	**Interpretation**		
	a)	Meaning: Give a cautious overall interpretation of results in the context of their limitations and in comparison with other studies	Our study advances the understanding of antihypertensive drugs by highlighting their potential to impact pain management significantly, far exceeding their conventional role in blood pressure regulation. This exploration suggests a paradigm shift in treating individuals with concurrent hypertension and pain disorders, advocating for an integrated approach that capitalizes on the multifaceted pharmacological effects of these medications. By identifying robust associations between antihypertensive drugs and a range of pain conditions, we underscore the necessity of holistic treatment strategies that consider the complex relationship between cardiovascular health and pain modulation.
	b)	Mechanism: Discuss underlying biological mechanisms that could drive a potential causal relationship between the investigated exposure and the outcome, and whether the gene-environment equivalence assumption is reasonable. Use causal language carefully, clarifying that IV estimates may provide causal effects only under certain assumptions	Migraine without aura involves intense headaches and sensory hypersensitivity, rooted in the trigeminovascular system's dysregulation affecting brain's vascular dynamics and inflammatory responses[26,44]. The sympathetic nervous system (SNS), which controls the body's 'fight or flight' response, also plays a pivotal role in vascular regulation by controlling vasoconstriction and vasodilation[33,36]. Adrenergic neuron blockers, by inhibiting SNS activity, could inadvertently modulate migraine pathophysiology in several interconnected ways. Firstly, these blockers induce vasodilation by reducing SNS-driven vasoconstriction. This vasodilation, particularly in cerebral vessels, can trigger the early phase of a

migraine attack, characterized by cortical spreading depression, neurogenic inflammation, and activation of pain pathways. The role of CGRP, a potent vasodilator released from sensory nerves, becomes crucial here[32]. CGRP levels and activity are closely regulated by the SNS, specifically via α2 adrenergic receptors. Studies have demonstrated that the SNS can influence sensory CGRP systems, indicating a complex interplay where SNS activity modulates CGRP release and function[21]. This interaction suggests that adrenergic neuron blockers, by dampening SNS activity, might lead to an imbalance in CGRP levels, potentially enhancing its vasodilatory and pro-inflammatory effects in the context of migraines. Moreover, research involving models of hypertension has shown that interventions affecting CGRP levels can alter the sensory nerve activation without directly influencing blood pressure, underscoring CGRP's role beyond vascular tone to include pain perception modulation[13]. The suppression of NGF-stimulated CGRP release via α2 receptor-mediated pathways and the observed effects of α2 antagonists in restoring CGRP levels further illustrate the intricate relationship between SNS signaling, CGRP regulation, and hypertension-related processes, which may mirror similar mechanisms in migraine pathogenesis[37].Therefore, the increased risk of migraine with aura associated with adrenergic neuron blockers might stem from these drugs' broad systemic effects on the SNS and CGRP regulation. This includes altering cerebral blood flow through vasodilation and disrupting the balance of neuropeptides crucial for pain and inflammatory responses, such as CGRP. The findings point toward a need for further investigation into the SNS-CGRP axis as a target for migraine therapies, especially considering the protective and triggering roles of CGRP in migraine pathophysiology[21].

Panniculitis, characterized by tender nodules and erythema within the subcutaneous fat layer, manifests from a variety of causes, ranging from infections to systemic diseases[27]. In contrast, loop diuretics, traditionally prescribed for controlling hypertension and fluid retention, act by inhibiting

sodium and chloride reabsorption in the Loop of Henle within the kidneys[25]. Our study introduces the speculative notion that loop diuretics might indirectly offer a novel therapeutic approach for managing panniculitis. By mitigating fluid retention, these medications could potentially alleviate the mechanical stress and inflammation in the subcutaneous fat layer, thereby providing symptomatic relief. Furthermore, the reduction in fluid overload might lead to a decrease in the body's overall inflammatory state, indirectly benefiting conditions characterized by inflammation, such as panniculitis. Enhanced circulation, a secondary effect of the action of loop diuretics, could improve the removal of inflammatory mediators from affected tissues and support the healing processes within the subcutaneous fat layer impacted by panniculitis. Incorporating these speculative mechanisms into our understanding of panniculitis treatment suggests that loop diuretics could potentially serve as an adjunct therapy, particularly in cases where fluid retention exacerbates the condition. However, empirical research is needed to validate these hypotheses and to determine whether loop diuretics could indeed offer a beneficial effect on the management of panniculitis.

Vasodilator antihypertensives offer a multifaceted approach to improving vascular health and alleviating limb pain, underscoring the intrinsic connection between vascular function and pain perception. These medications enhance blood flow and reduce vascular resistance through several possible key mechanisms: enhancing nitric oxide availability for endothelial function improvement, directly inducing vasodilation, blocking endothelin receptors to counteract vasoconstriction, activating the prostacyclin pathway to improve microcirculation and inhibit thrombosis, and increasing perfusion pressure in compromised vascular territories. Such actions not only decrease peripheral resistance but also ensure a better blood supply to ischemic regions, directly addressing the pain associated with inadequate circulation. This improved blood flow is critical for relieving pain in conditions where ischemia or poor circulation is a contributing factor. By relaxing vascular smooth

muscle and enhancing endothelial responses, these drugs mitigate the mechanical stress on blood vessels and support the delivery of oxygen and nutrients to affected tissues. The blockade of endothelin receptors and activation of prostacyclin pathways further contribute to vasodilation, offering additional routes to counteract the effects of vasoconstriction that exacerbates pain. The potential application of vasodilator antihypertensives in future treatment strategies for limb pain is promising, given their capacity to address the underlying vascular issues contributing to pain.

Our analysis also sheds light on suggestive evidence necessitating further investigation. Notably, the increased risk of femgenpain (Pain and other conditions associated with female genital organs and menstrual cycle) associated with adrenergic neuron blockers raises concerns about their use in conditions related to female reproductive health, suggesting a complex interaction with the SNS that warrants cautious application. Moreover, our observations on vasodilator antihypertensives suggest their utility in alleviating radiculopathy, further advocating for the exploration of their role in conditions characterized by nerve root irritation or compression. The tentative evidence surrounding alpha-adrenoceptor blockers and their dichotomous impact on conditions like dorsalgia and migraines, along with renin inhibitors' potential protective effect against dorsalgianas, underscores the complex interplay between cardiovascular pharmacology and pain modulation. These findings prompt a comprehensive exploration into the cardiovascular system's role in pain pathology and the therapeutic potential of antihypertensive drugs in pain management.

While previous studies have established BBs as effective in reducing perioperative pain and minimizing long-term opioid usage[29,42], our findings introduce a novel perspective by identifying their protective role against radiculopathy. While previous research has touted the efficacy of BBs in mitigating OA symptoms, particularly in knee and hip joints, our study presents a divergent narrative. Our results do not

support the effectiveness of BBs in OA management, instead highlighting the protective effects of adrenergic neuron blockers against coxarthrosis. By enhancing joint microcirculation and reducing inflammation, these drugs may present a novel strategy for managing OA pain, underscoring the need for targeted research to validate these findings.

Our research contributes to the complex discourse on the role of CCBs in pain management by revealing no significant association between CCBs and pain disorders. This finding contrasts sharply with previous studies suggesting their effectiveness in treating headaches[40]. This discrepancy highlights the nuanced and multifaceted nature of pain modulation and the challenges inherent in repurposing CCBs for pain management. While biological rationales and their application in migraine treatment hint at a potential connection to pain conditions, the evidence from extensive human studies remains divided[30]. Notably, some research points to worsened pain outcomes in conditions like knee OA (KOA) with CCB use, whereas other studies hint at potential analgesic properties[24,41]. These mixed outcomes underscore the necessity for a more discerning approach to employing CCBs in pain management, emphasizing the importance of further targeted research. Such studies are crucial for disentangling the beneficial from the potentially adverse effects of calcium channel modulation on various pain pathways.

c) Clinical relevance: Discuss whether the results have clinical or public policy relevance, and to what extent they inform effect sizes of possible interventions

Our study advances the understanding of antihypertensive drugs by highlighting their potential to impact pain management significantly, far exceeding their conventional role in blood pressure regulation. This exploration suggests a paradigm shift in treating individuals with concurrent hypertension and pain disorders, advocating for an integrated approach that capitalizes on the multifaceted pharmacological effects of these medications. By identifying robust associations between antihypertensive drugs and a range of pain conditions, we underscore the necessity of holistic treatment strategies that consider the complex relationship between cardiovascular health and pain modulation.

			Our exploration of antihypertensive drugs for pain management uncovers novel therapeutic possibilities and signifies a critical shift towards a personalized medicine approach for comorbid hypertension and pain. By tailoring treatments to individuals' genetic profiles and the specific pharmacodynamics of these drugs, we anticipate substantial improvements in managing both conditions and enhancing patient outcomes. This direction necessitates further validation through targeted clinical trials, comprehensive longitudinal studies to assess long-term effects, and fundamental scientific research to elucidate molecular mechanisms, ensuring that our findings effectively inform and refine clinical guidelines.
17	**Generalizability**	Discuss the generalizability of the study results (a) to other populations, (b) across other exposure periods/timings, and (c) across other levels of exposure	While our study employs a robust MR framework to investigate the role of antihypertensive drugs in pain management, the reliance on genetic data predominantly from populations of European ancestry limits the generalizability of our findings.
	**OTHER INFORMATION**		
18	**Funding**	Describe sources of funding and the role of funders in the present study and, if applicable, sources of funding for the databases and original study or studies on which the present study is based	This study was supported by Capital's Funds for Health Improvement and Research (No.2020-2-2231).
19	**Data and data sharing**	Provide the data used to perform all analyses or report where and how the data can be accessed, and reference these sources in the article. Provide the statistical code needed to reproduce the results in the article, or report whether the code is publicly accessible and if so, where	All data used in this study were obtained from IEU Open GWAS (https://gwas.mrcieu.ac.uk/).
20	**Conflicts of Interest**	All authors should declare all potential conflicts of interest	A statement was made by the authors that this study was conducted without any business or financial relationships that could be perceived as a potential conflict of interest.

This checklist is copyrighted by the Equator Network under the Creative Commons Attribution 3.0 Unported (CC BY 3.0) license.

1. Skrivankova VW, Richmond RC, Woolf BAR, Yarmolinsky J, Davies NM, Swanson SA, et al. Strengthening the Reporting of Observational Studies in Epidemiology using Mendelian Randomization (STROBE-MR) Statement. JAMA. 2021;under review.

2. Skrivankova VW, Richmond RC, Woolf BAR, Davies NM, Swanson SA, VanderWeele TJ, et al. Strengthening the Reporting of Observational Studies in Epidemiology using Mendelian Randomisation (STROBE-MR): Explanation and Elaboration. BMJ. 2021;375:n2233.
