# Supplementary material for: Repurposing antihypertensive drugs for pain disorders: a drug-target mendelian randomization study
Source: Front Pharmacol. 2024 Aug 29;15:1448319. doi: 10.3389/fphar.2024.1448319 (PMC11390634; doi:10.3389/fphar.2024.1448319)
Supplement: Supplementary file 3 [file DataSheet1.docx]

***Repurposing Antihypertensive Drugs for Pain Disorders: A Drug-Target Mendelian Randomization Study***

***Kai Du^1^, Ao Li^1^, Chen-Yu Zhang ^1^, Shu-Ming Li ^2, *^, Ping Chen ^2, *^***


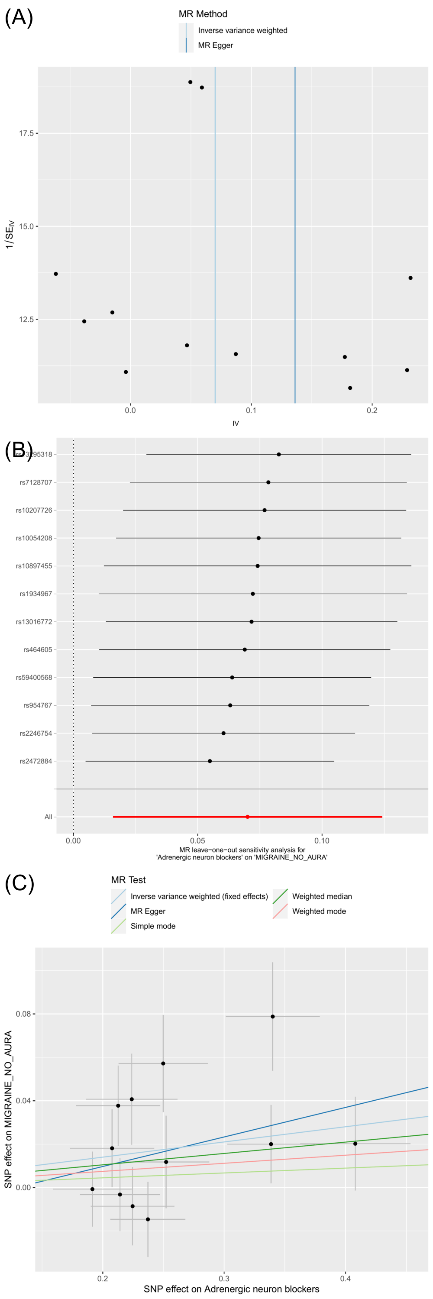


**Supplementary Figure 1.** Mendelian Randomization Analysis of the Causal Relationship between Exposure (Adrenergic neuron blockers) and Outcome (MIGRAINE_NO_AURA). A: Funnel plot; B: Leave-one-out stability tests; C: Scatter plot.

**
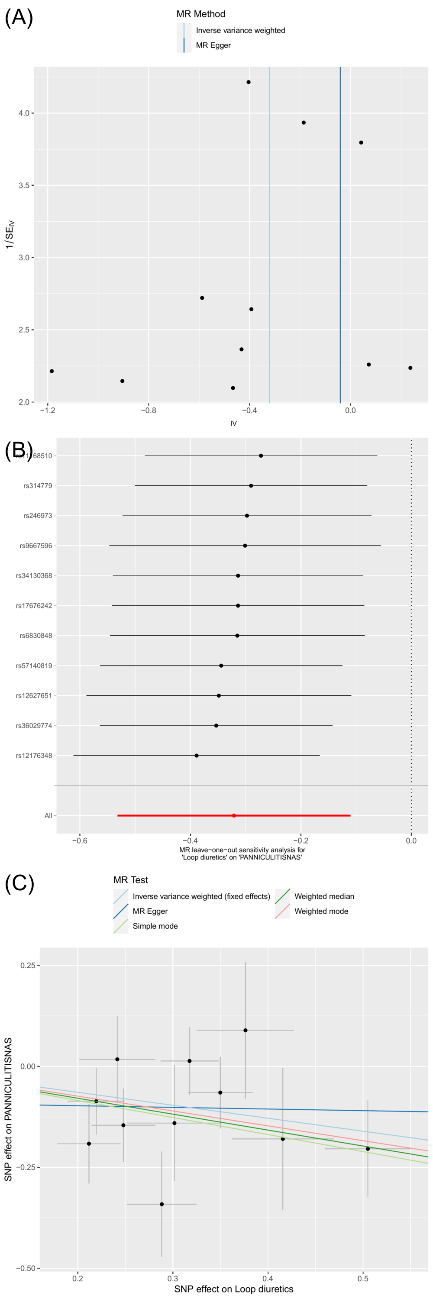
**

**Supplementary Figure 2.** Mendelian Randomization Analysis of the Causal Relationship between Exposure (Loop diuretics) and Outcome (PANNICULITISNAS). A: Funnel plot; B: Leave-one-out stability tests; C: Scatter plot.

**
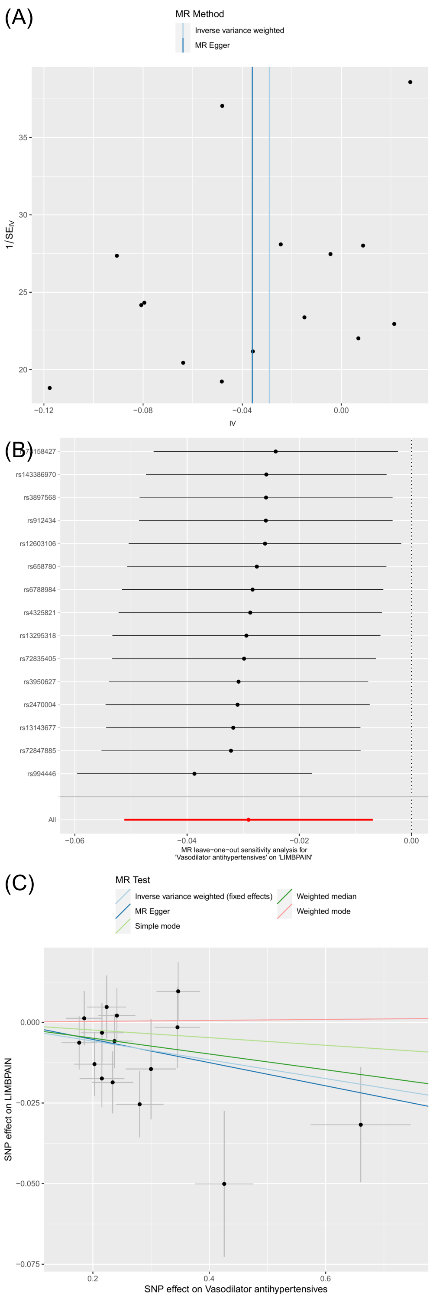
**

**Supplementary Figure 3.** Mendelian Randomization Analysis of the Causal Relationship between Exposure (Vasodilator antihypertensives) and Outcome (LIMBPAIN). A: Funnel plot; B: Leave-one-out stability tests; C: Scatter plot.


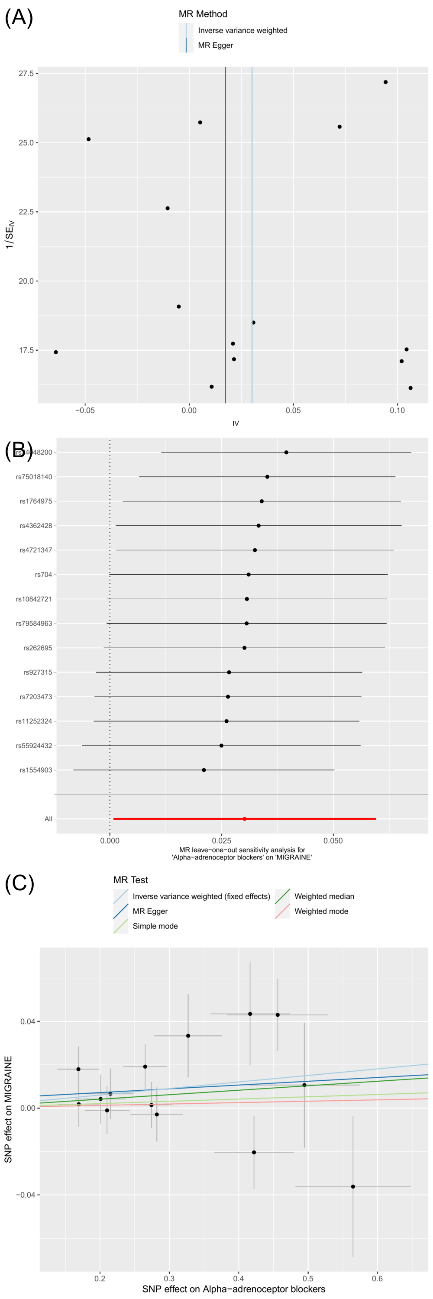


**Supplementary Figure 4.** Mendelian Randomization Analysis of the Causal Relationship between Exposure (Alpha−adrenoceptor blockers) and Outcome (MIGRAINE). A: Funnel plot; B: Leave-one-out stability tests; C: Scatter plot.


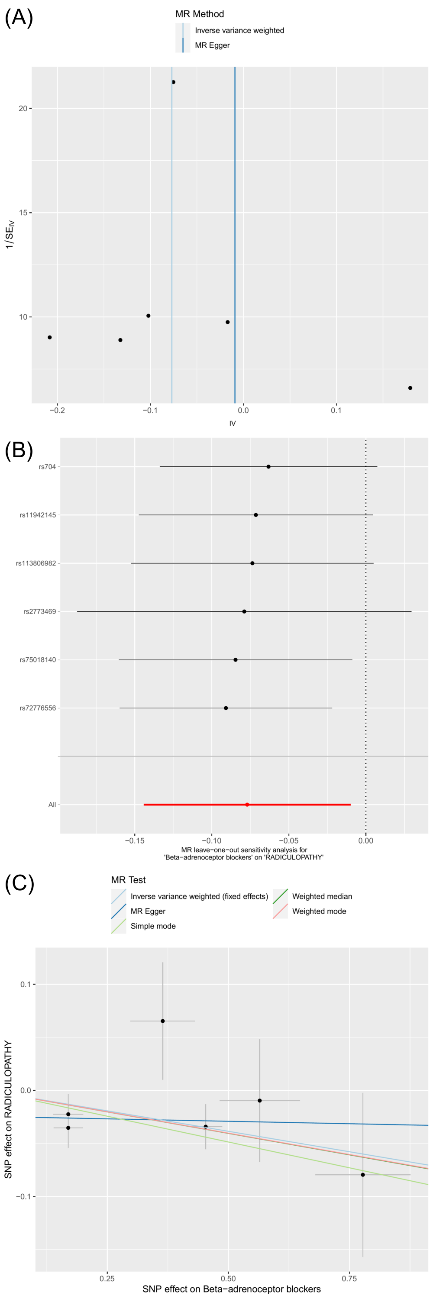


**Supplementary Figure 5.** Mendelian Randomization Analysis of the Causal Relationship between Exposure (Beta−adrenoceptor blockers) and Outcome (RADICULOPATHY). A: Funnel plot; B: Leave-one-out stability tests; C: Scatter plot.

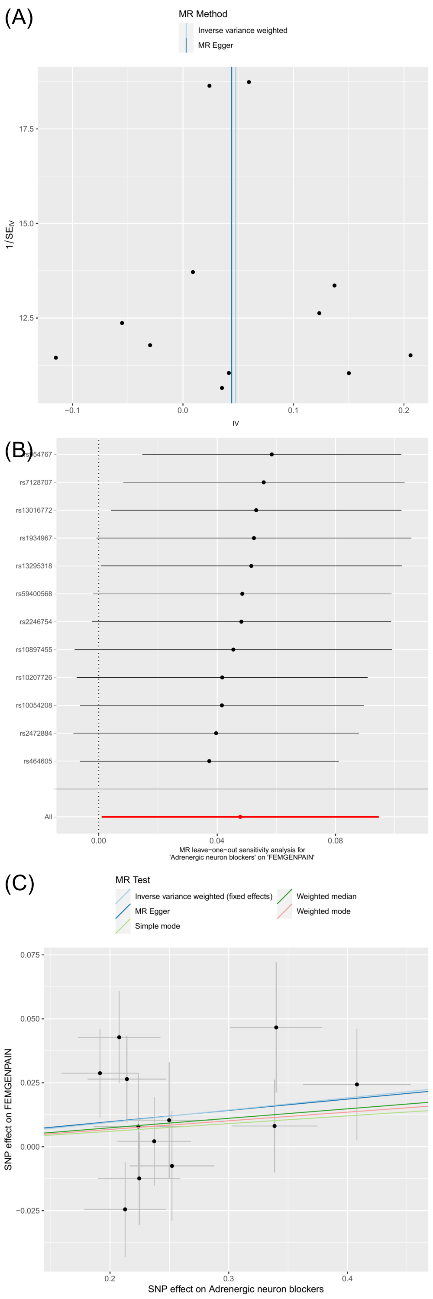


**Supplementary Figure 6.** Mendelian Randomization Analysis of the Causal Relationship between Exposure (Adrenergic neuron blockers) and Outcome (FEMGENPAIN). A: Funnel plot; B: Leave-one-out stability tests; C: Scatter plot.

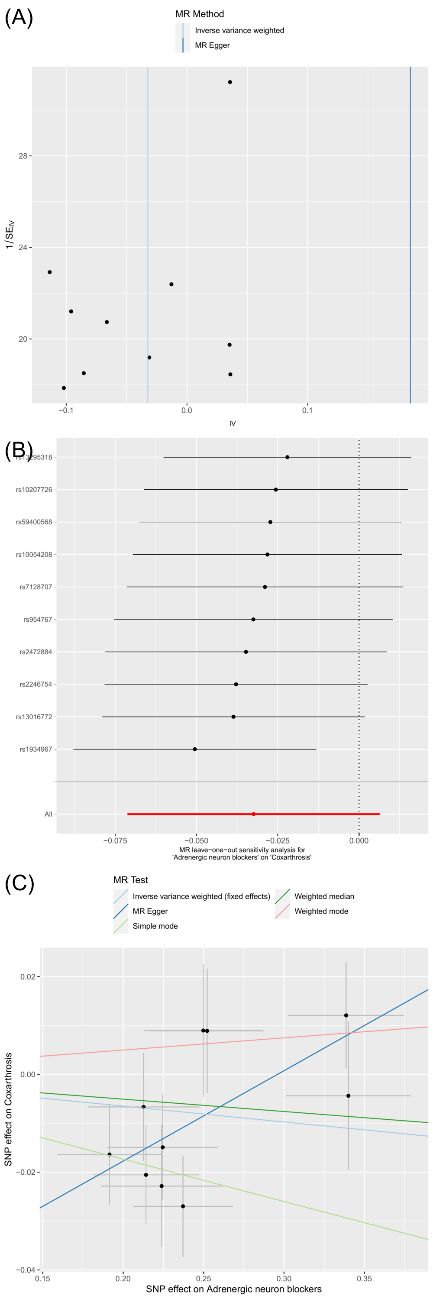


**Supplementary Figure 7.** Mendelian Randomization Analysis of the Causal Relationship between Exposure (Adrenergic neuron blockers) and Outcome (Coxarthrosis). A: Funnel plot; B: Leave-one-out stability tests; C: Scatter plot.

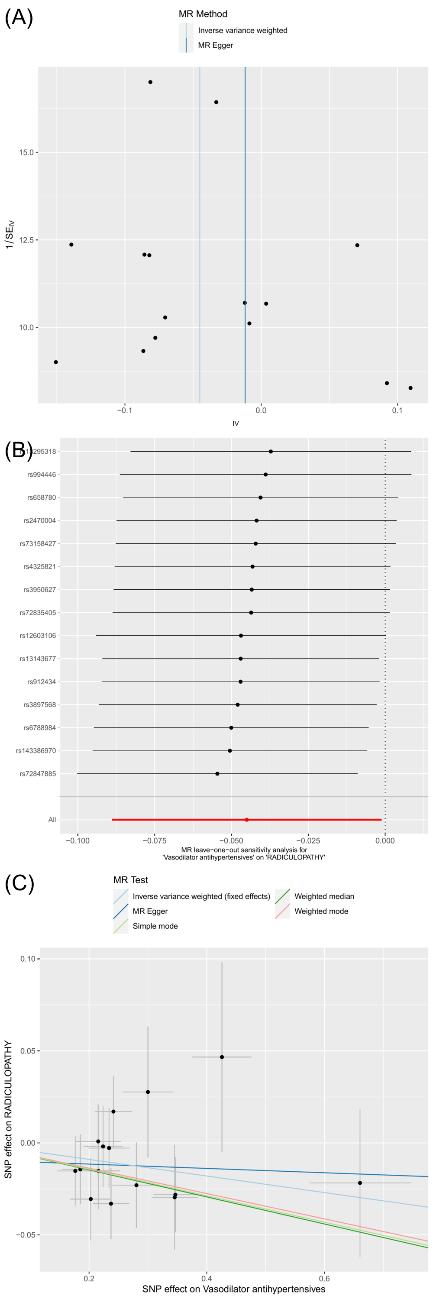


**Supplementary Figure 8.** Mendelian Randomization Analysis of the Causal Relationship between Exposure (Vasodilator antihypertensives) and Outcome (RADICULOPATHY). A: Funnel plot; B: Leave-one-out stability tests; C: Scatter plot.


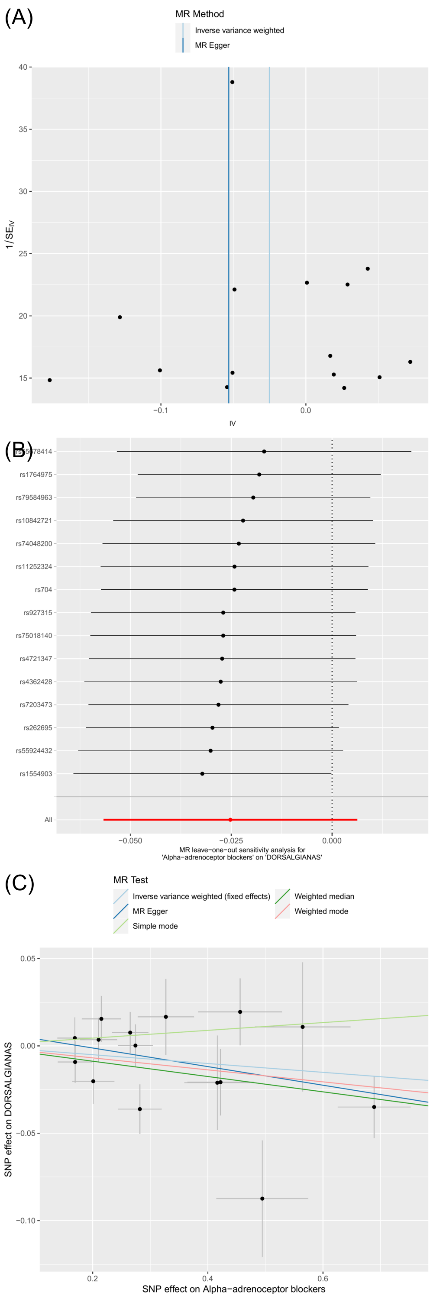


**Supplementary Figure 9.** Mendelian Randomization Analysis of the Causal Relationship between Exposure (Alpha−adrenoceptor blockers) and Outcome (DORSALGIANAS).
A: Funnel plot; B: Leave-one-out stability tests; C: Scatter plot.
